# Single Ni Atoms Drive Carboxyl Deprotonation in Metal–Organic Chains

**DOI:** 10.1021/acsnano.6c01444

**Published:** 2026-04-17

**Authors:** Simone Mearini, Fabian Auer, Maximilian Laßhofer, Andreas Windischbacher, Dominik Brandstetter, Daniel Baranowski, Yan Yan Grisan Qiu, Iulia Cojocariu, Matteo Jugovac, Martin Sterrer, Giovanni Zamborlini, Vitaliy Feyer, Claus Michael Schneider

**Affiliations:** † Peter Grünberg Institute (PGI-6), Jülich Research Centre, 52428 Jülich, Germany; ‡ Institute of Physics NAWI Graz, University of Graz, 8010 Graz, Austria; § Physics Department, 9315 University of Trieste, 34127 Trieste, Italy; ∥ Elettra—Sincrotrone Trieste S.C.p.A., S.S. 14 km 163.5, 34149 Trieste, Italy; ⊥ Faculty of Physics and Center for Nanointegration Duisburg-Essen (CENIDE), University of Duisburg-Essen, 47048 Duisburg, Germany; # Department of Physics and Astronomy, UC Davis, Davis California 95616, United States

**Keywords:** molecular self-assembly, linear metal−organic chains, Ni(I) oxidation state, deprotonation, orbital hybridization

## Abstract

Understanding how charge transfer and ligand activation processes govern metal–organic coordination at surfaces is crucial for controlling on-surface synthesis and the formation of low-dimensional architectures. Here, we show that Ni promotes the deprotonation of the carboxyl groups of terephthalic acid (TPA) on Ag(100), leading to the formation of linear metal–organic coordination chains. Scanning tunneling microscopy reveals that these chains emerge from preassembled hydrogen-bonded TPA stripes. X-ray photoelectron spectroscopy identifies stabilization of the Ni centers in the Ni­(I) oxidation state through a single-electron charge transfer process, accompanied by the formation of deprotonated carboxylate species. Valence band spectroscopy reveals a coordination-induced electronic reorganization between Ni and TPA through the emergence of hybrid states, in agreement with complementary theoretical modeling. Together, these findings identify charge-transfer-driven deprotonation as the central mechanism governing the formation of linear metal–organic chains.

## Graphical abstract

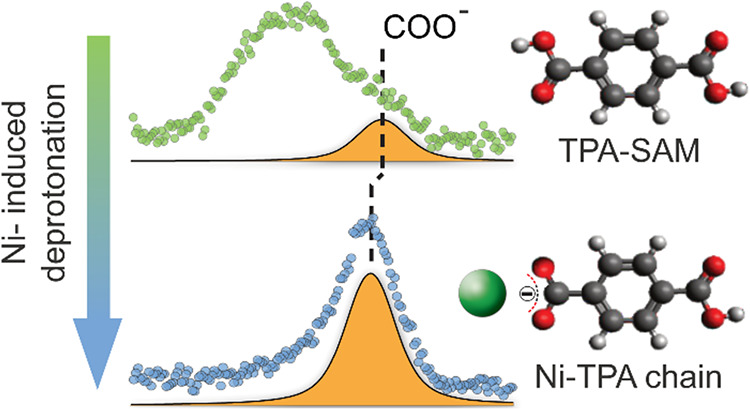

## Introduction

The on-surface synthesis of atomically thin metal–organic architectures offers a versatile route to create ordered assemblies with tunable structural and electronic properties at the nanoscale.^1–12^ In this respect, substrate–adsorbate interactions, ligand electron affinity, and metal–ligand coordination determine the arrangement of these assemblies. By controlling these factors, it is possible to form discrete metal–organic complexes,^13–17^ linear coordination motifs,^18–23^ or extended two-dimensional metal–organic frameworks (2D MOFs).^8,13,17,24–27^ This flexibility not only allows control over the structure, but also enables the stabilization of specific metal oxidation states and electronic configurations.^24,28,29^

A prototypical building block for obtaining such complex molecular assemblies is terephthalic acid (TPA).^30–33^ TPA is a dicarboxyl ligand, in which the two para-positioned carboxyl (−COOH) groups can lead to different coordination motifs.^14,33–35^ Upon deposition on metal substrates, TPA can self-assemble into linear stripes stabilized by –C–OH···O=C hydrogen-bond interactions,^30,33,36^ or undergo deprotonation of one or both carboxyl groups to carboxylates, −COO^–^.^14,33,37,38^ The latter behavior is more commonly observed on strongly interacting substrates, where ligand activation is promoted by enhanced molecular-surface interactions.

In surface-supported 2D MOFs, deprotonated TPA species readily incorporate metal centers. For example, in combination with holmium (Ho) atoms, a Ho-TPA network forms on Ag(100) after complete deprotonation of the carboxyl groups is achieved by deposition onto a preheated substrate.^33^ In this case, the metal centers bind to preactivated ligands rather than driving ligand activation. A similar behavior has been observed for Fe-TPA^14^ and Dy-TPA^39^ networks. Importantly, in all previous studies, MOF formation has relied on intentional predeprotonation of the TPA ligand before metal incorporation.

However, substrate adatoms of Ni(111) and other transition metal (TM) surfaces have already shown the ability to activate the carboxyl functional groups of related molecular systems.^40^ This raises the question of whether single adsorbed metal atoms can directly promote the ligand deprotonation during incorporation, without the need for prior substrate-induced activation.

For the metal center, we select Ni which has been shown to form extended 2D MOFs on surfaces with molecules such as 9,9,10,10-tetracyanoquinodimethane (TCNQ) and 1,2,4,5-tetracyanobenzene (TCNB).^13,17,24,41^ For the initial molecular layer, TPA is deposited on Ag(100) at room temperature, yielding a characteristic structure based on hydrogen-bonded linear stripes, as observed by scanning tunneling microscopy (STM). Upon annealing and subsequent Ni deposition, the overall molecular arrangement remains largely preserved, indicating minimal structural rearrangements. Valence band (VB) spectroscopy reveals new electronic states near the Fermi level, absent in both the pristine and annealed TPA self-assembled monolayer (SAM). These features are consistent with Ni-ligand hybridization and indicate the formation of extended coordination structures.^24,42^

Finally, XPS provides direct insights into the chemical processes accompanying Ni coordination. The pristine TPA SAM exhibits only partial deprotonation due to the hydrogen-bond stabilization within the chains. Ni deposition, however, actively promotes further deprotonation of the carboxyl groups via single-electron donation, stabilizing the metal centers in the Ni­(I) oxidation state and enabling the Ni-ligand bond formation.

Altogether, these results demonstrate how single-atom coordination and charge redistribution within a predefined linear molecular template govern on-surface metal–organic assembly and determine both the structural motif and the resulting electronic properties in low-dimensional systems. Complementary density functional theory calculations further support this picture by providing insight into the electronic structure, charge redistribution, and coordination environments of the Ni-TPA system.

## Results and Discussion

TPA molecules, whose model structures in the protonated and deprotonated conformations are depicted in Figure 1a,b, respectively, are sublimated onto an Ag(100) crystal held at room temperature. Figure 1c shows the STM image of an ordered SAM with linearly arranged molecules in a stripe-like pattern. The large-scale image reported in Figure S1a (Supporting Information) confirms the long-range order of this arrangement. In the following, this structure is referred to as the “α-phase”, whose unit cell is defined by the two lattice vectors *a*_1_ = *a*_2_ = *a* = 7.6 ± 0.3 Å and an enclosed angle ϑ = 81 ± 1°, forming a supercell with the Ag(100) substrate described by the matrix (2, 1.7; 2, −1.7). The lattice vector *a*_1_ encloses an angle of ∼40° with respect to the [01̅1] direction of the substrate. The LEED pattern (Figure S2a), together with its simulation using the matrix above, confirms this structural assignment.

**Figure 1:**
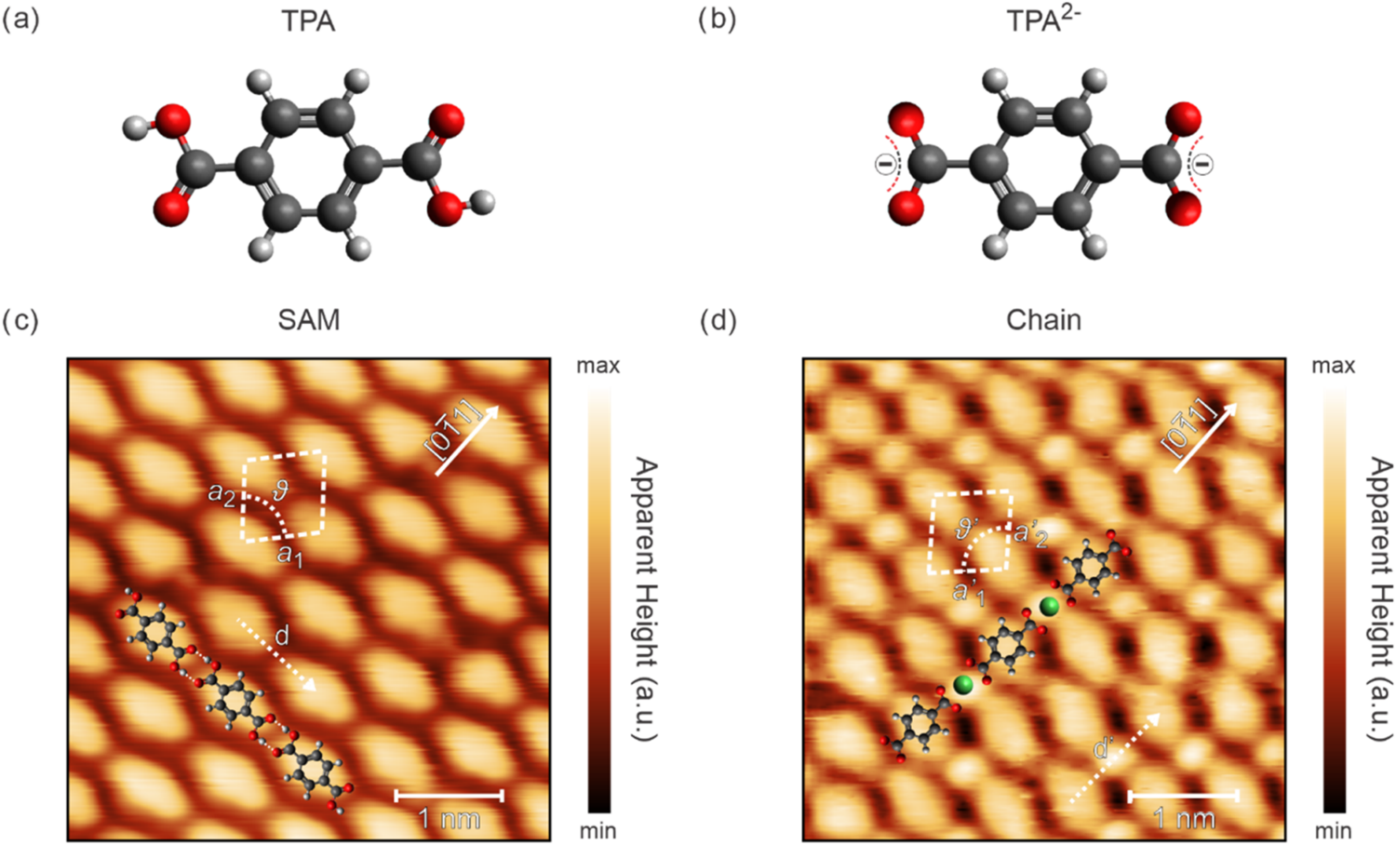
Chemical structures of (a) protonated TPA and (b) doubly deprotonated TPA^2–^; STM images of (c) the SAM “α-phase” (4.5 × 4.5 nm^2^, −3 V, 0.5 nA) and (d) the Ni-TPA chains (4.5 × 4.5 nm^2^, −0.3 V, 0.5 nA). The images show different rotational domains. The unit cells are indicated by white dashed lines, while the intrastripe distances *d* and *d*′ are marked by white dotted arrows. A model of the molecular arrangement is shown overlaid on both images. In (c), dashed lines within the model indicate the hydrogen-bonding interactions.

In this configuration, the centers of two consecutive molecules within the same stripe are separated by a distance *d* = 9.7 ± 0.3 Å, which is in good agreement with the value reported in the literature.^33^ The elongated TPA shape indicates alignment of the carboxyl (−COOH) groups along the stripes, supposedly with adjacent −COOH groups forming double hydrogen-bonds.^33^ From the molecular geometry observed in the STM image (Figure 1c), the distance between adjacent oxygen atoms (O···O) involved in the hydrogen-bonded –C–OH···O=C motifs is estimated to be approximately 2.7 ± 0.3 Å. This distance is in good agreement with reported O···O distances for hydrogen-bonded TPA assemblies on coinage metal surfaces.^30,33,36,43^ These directional interactions stabilize the observed linear arrangement.

Uphoff et al.^33^ have reported that TPA deposition on a preheated Ag(100) substrate (∼450 K) results in a near complete deprotonation of the −COOH groups, forming a more compact phase denoted as the “β-phase”. In our STM measurements of the pristine α-phase, formed by TPA deposition at room temperature, neither the β-phase nor any thermally induced structural rearrangements have been observed, even after postannealing up to 363 K, which is well below the molecular desorption temperature from the Ag(100) substrate in this phase (∼400 K). We therefore conclude that the α-phase remains stable under the conditions studied, and that the β-phase is not accessible within the experimental approach explored in this work.

Based on these findings, the TPA self-assembled monolayer was annealed to 363 K prior to Ni deposition to improve long-range molecular ordering. Ni was then deposited with the substrate held at 363 K, a temperature chosen to promote surface diffusion of Ni adatoms, while remaining below the molecular desorption threshold. Under these conditions, upon Ni intrusion, the α-phase undergoes minor structural rearrangements while still preserving its linearity. STM images (Figure 1d and S1b, Supporting Information) show new features appearing in the intrastripe spaces between molecules where hydrogen-bonds were previously located. Accordingly, the intrastripe distance *d*′ = 11.0 ± 0.3 Å (indicated by the dotted line in Figure 1d) is larger than that of the pristine α-phase (*d* = 9.7 ± 0.3 Å). The primitive unit cell (Figure 1d) is characterized by *a*′_1_ = *a*′_2_ = *a*′= 7.4 ± 0.3 Å, and ϑ′ = 96 ± 1°, corresponding to the incommensurate matrix (1.9, 1.7; 1.9, −1.7). The lattice vector *a*′_1_ forms an angle of ∼42° relative to the [01̅1] direction of Ag(100). These parameters are corroborated by the experimental and simulated LEED results reported in Figure S2b.

Our results suggest the formation of linear metal–organic chains (MOCs), in which Ni atoms bridge neighboring ligands along a single direction. This structural motif contrasts with that observed in Ho-TPA/Ag(100)^33^ and other well-studied systems, such as Fe-TPA/Cu(100),^14,44,45^ where metal coordination drives the formation of extended 2D MOFs with coordinative bonds extending along both in-plane directions.

While the emerging bright protrusions between TPA molecules observed in STM are strongly indicative of Ni-incorporation, Ni-TPA coordination can be confirmed by electronic structure analysis. To this end, momentum-resolved photoemission measurements of the VB region have been performed. Momentum-integrated spectra (Figure 2a,c) and corresponding band maps (Figure 2b,d) are extracted from the three-dimensional (*k*_*x*_, *k*_*y*_, BE) data set, where BE denotes the binding energy. The band maps are reported as momentum cuts along the *M̅*–Γ̅–*X̅* direction of the Ag(100) surface Brillouin zone, as indicated in the momentum map of the clean Ag(100) surface in Figure S3. Results for the annealed SAM and the clean Ag(100) substrate are provided in Figure S4 (Supporting Information).

**Figure 2:**
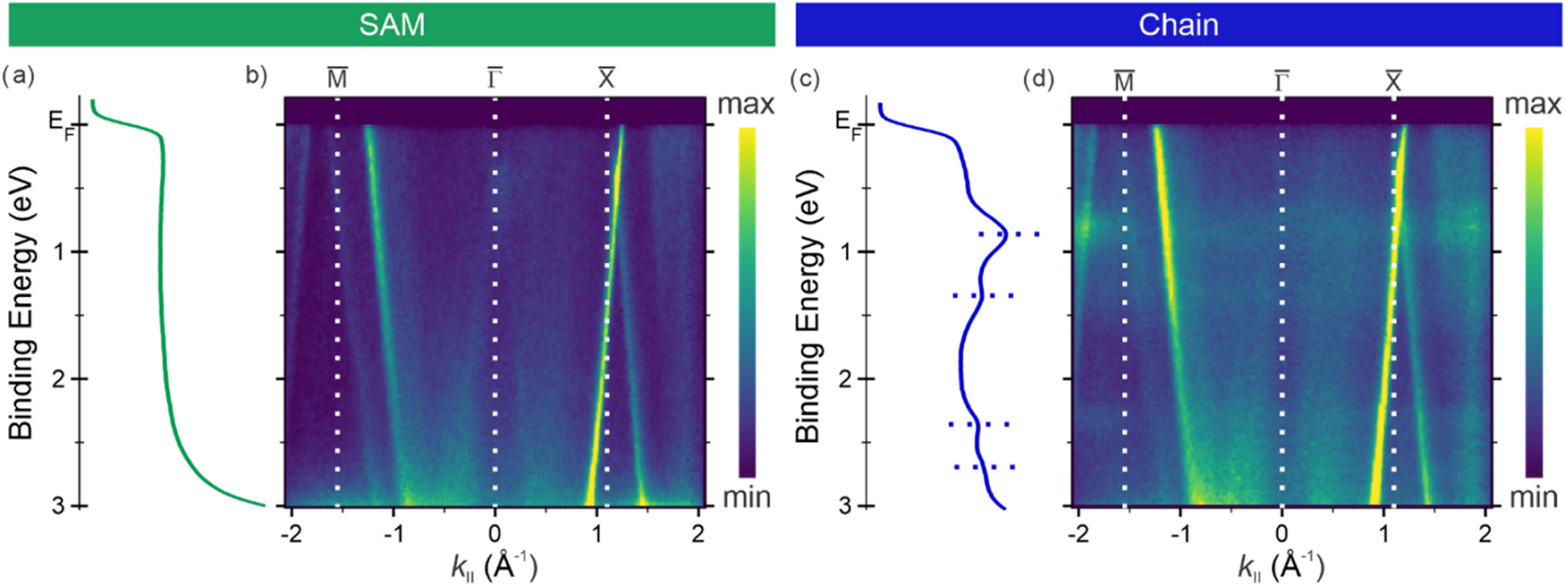
(a, c) Momentum-integrated valence band spectra and (b, d) band maps (*h*ν = 30 eV, p-polarized light) for (a, b) the SAM and (c, d) the Ni-TPA chains in the [–0.2, 3.0] eV BE range. Band maps show BE vs momentum cuts along the *M̅* −Γ̅–*X̅* direction of Ag(100). In (c), dotted lines highlight hybrid Ni-TPA features.

The VB spectrum and band map of the SAM (Figure 2a,b) are featureless up to 2.9 eV. Molecular features appear at higher BE and overlap with the Ag 4d band, as shown in Figure S4. These features are attributed to the highest occupied molecular orbital (HOMO) and higher-energy molecular states of TPA. This interpretation is supported by density functional theory (DFT) calculations (further details in Supporting Information, Theoretical Models section), which indicate the absence of significant charge transfer between the molecular adlayer and the substrate.

While the SAM annealing induces only minor changes in the VB (Figure S4), Ni incorporation leads to pronounced modifications (Figure 2c,d). New electronic states emerge between the Fermi level and 3 eV in the Ni-TPA chains, which are absent in the pristine annealed SAM. The energy positions of these states (Figure 2c,d) align with hybrid features previously reported for TCNB-based complexes and 2D Ni-TCNB MOFs,^13,24^ supporting their assignment to Ni-ligand hybrid states and showing good agreement with DFT simulations of the Ni-TPA interface (Supporting Information, Theoretical Models section).

To probe the chemical state of both ligands and Ni in this linear coordination environment, XPS measurements have been performed on the pristine SAM and the Ni-TPA MOC, focusing on the O 1s (Figures 3a and S5) and Ni 2p (Figures 3b and S7) core-level regions.

**Figure 3:**
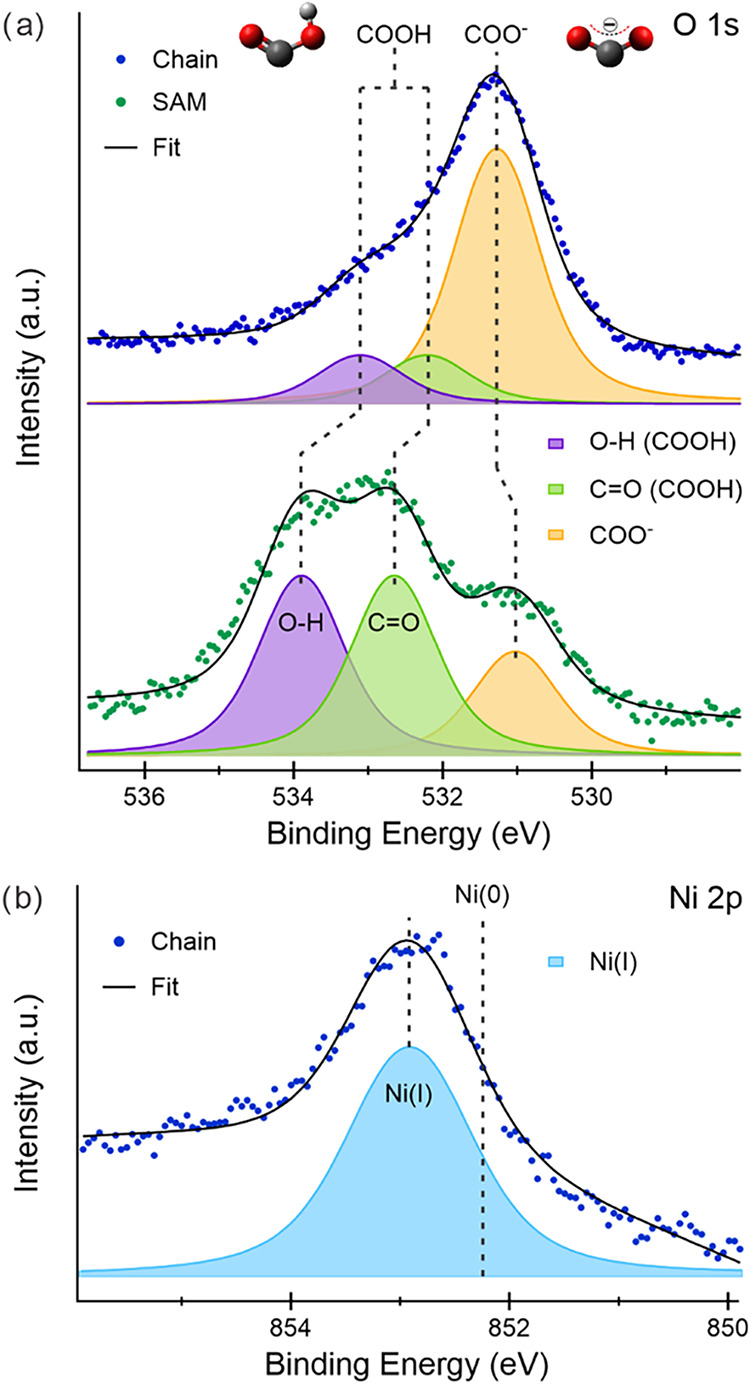
(a) O 1s XPS spectra (dots) and corresponding fits (black curves) using Voigt functions of the SAM (green) and Ni-TPA chains (blue); the deconvolution components are reported in purple and light green for the O–H and C=O oxygen atoms of the −COOH groups, respectively, and in orange for the COO^–^ oxygen atoms in TPA^2–^. (b) Ni 2p XPS spectrum (0.08 ML, blue dots) and corresponding fit (black curve) using Voigt functions of the Ni-TPA chains; the cyan component corresponds to the deconvoluted contribution of Ni­(I) atoms, while the “Ni(0)” label indicates the binding energy position of a reference layer.

For the annealed SAM reference, the TPA layer was heated to 363 K prior to Ni deposition in order to quantify the temperature-induced deprotonation in the absence of Ni and to enable a direct comparison with Ni-induced deprotonation under identical thermal conditions. The spectral fitting results are summarized in Table S1, with details on the adopted procedure provided in the Experimental section.

In the O 1s spectra (Figure 3a), clear differences are evident between the SAM (green dots) and the Ni-TPA chains (blue dots). The SAM spectrum exhibits three contributions, consistent with previous reports for a TPA monolayer on Sn/Cu(100),^32^ while the Ni-TPA spectrum is dominated by a single main feature with a weak shoulder at higher BE.

For the SAM, the O 1s peaks centered at 532.6 and 533.8 eV are assigned to the carbonyl (C=O) and hydroxyl (O–H) oxygen atoms of the protonated TPA, respectively.^32^ Their comparable areas reflect the 1:1 stoichiometry of oxygen atoms in −COOH groups. The energy separation (ΔBE = 1.2 eV) is consistent with the presence of mostly double hydrogen-bonds between adjacent −COOH groups.^32^ The lower BE peak, centered at 531.0 eV, is attributed to the two equivalent oxygen atoms of a deprotonated carboxylate (−COO^–^). A quantitative analysis (Table S1) indicates that protonated and deprotonated groups coexist in the SAM with a strong majority of the former species, supporting the linearly organized stripe pattern observed in STM (Figure 1c).

The small fraction of isolated deprotonated molecules can form only a single hydrogen-bond with neighboring molecules or interact strongly with the substrate as suggested by DFT calculations (Supporting Information, Theoretical Models section), creating local defects. STM images indeed reveal sporadic irregularities in molecular shapes along the hydrogen-bond direction (Figure S6), consistent with the presence of such defects. However, these defects do not significantly affect the overall linear arrangement of the network.

Annealing the SAM to 363 K (Figure S5) preserves all three components in the O 1s spectrum, with altered relative intensities. At higher annealing temperatures (>400 K), the supramolecular order shown in Figure S2a is lost due to desorption, as confirmed by the recovery of the substrate’s LEED spots. These observations indicate that the Ag(100)-induced deprotonation in the SAM α-phase is limited under the present conditions. In contrast, the SAM β-phase, obtained by deposition of TPA onto a substrate held at 450 K,^33^ is dominated by TPA^2–^ species. This comparison highlights the role of substrate temperature and hydrogen-bond stabilization in controlling ligand activation.

For the Ni-TPA chains, the O 1s spectrum (dark blue dots, Figure 3a) is dominated by a single peak centered at 531.2 eV, assigned to carboxylate groups coordinated to Ni centers. This peak is shifted by +0.2 eV relative to the SAM (531.0 eV), consistent with a chemical shift induced by Ni-ligand coordination, as reported for related metal–organic systems.^14^ The weak shoulder at higher BE, which can be deconvoluted into two components centered at 533.1 and 532.2 eV, indicates residual protonated species forming single hydrogen-bonds, in agreement with previous reports for TPA/Cu(100).^32^

The Ni 2p spectrum (Figure 3b) provides direct information on the oxidation state of the metal centers. A reference film of Ni deposited directly on Ag(100) (black dots, Figure S7) exhibits a characteristic Ni(0) component, whereas the spectrum of the Ni-TPA (dark blue dots, Figures 3b and S7) shows a +0.7 eV BE shift. This shift is consistent with stabilization of Ni centers in the Ni­(I) oxidation state,^13,46,47^ in agreement with DFT simulations of the Ni-TPA interface. Analysis of the calculated density of states (Supporting Information, Theoretical Models section) indicates depletion of Ni-derived states and charge transfer toward the ligands, further supporting Ni­(I) stabilization.

Based on these results, we now discuss the deprotonation process associated with the formation of the Ni-TPA MOC. To support this analysis, Figure 4 summarizes the O 1s XPS spectra of the freshly prepared SAM, the SAM annealed to 363 K, and the Ni-saturated Ni-TPA chains. The relative contributions of the carboxyl species have been quantified (Table S2), and the corresponding deprotonation fractions are presented in Figure 4.

**Figure 4:**
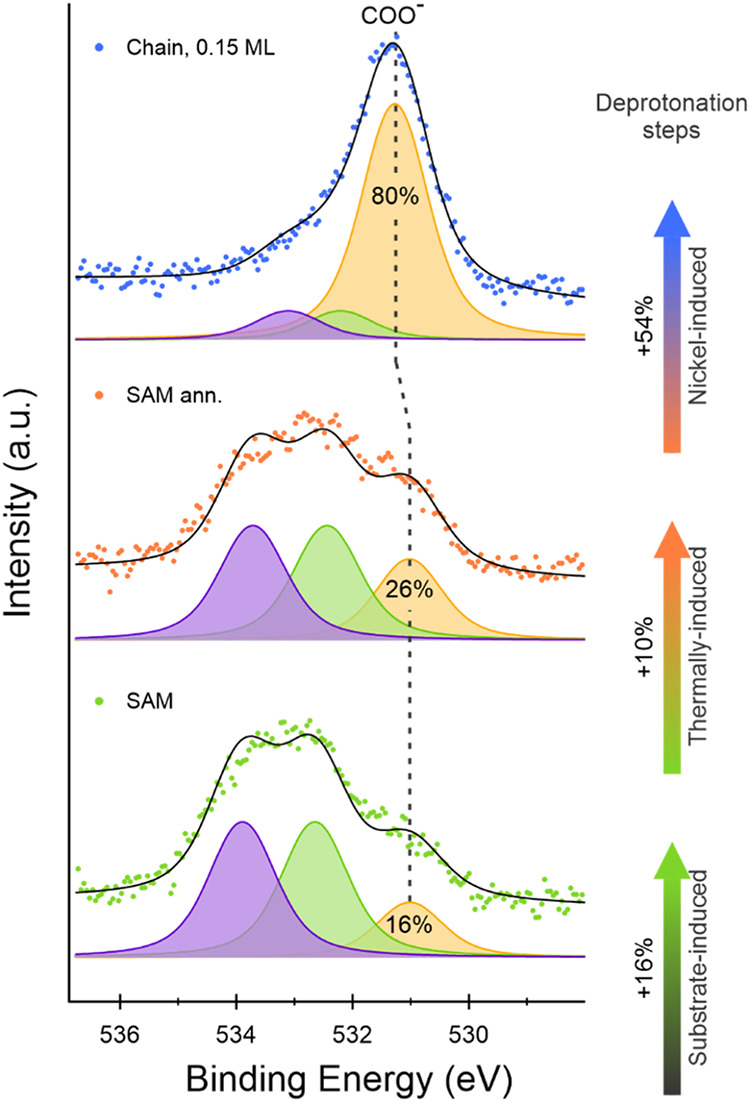
O 1s XPS spectra (dots) and corresponding fits (black curves) using Voigt functions for (a) the freshly prepared SAM, (b) the annealed layer (*T* = 363 K), and (c) the Ni-TPA chains with 0.15 ML of Ni; the deconvolution is reported in purple and light green for the O–H and C=O oxygen atoms from the −COOH groups, respectively, and in orange for the COO^–^ oxygen atoms in TPA^2–^.

The pristine SAM exhibits a protonated-to-deprotonated ratio of approximately 4:1, corresponding to 16% COO^–^ groups in the layer. Upon annealing, the deprotonated fraction increases to 26% (ratio ∼ 3:1), indicating that Ag(100) alone can activate only a limited fraction of the carboxyl groups. This activation is illustrated in the first step of Scheme 1. Since the VB measurements indicate no detectable charge transfer between the molecules and the substrate (Figure 2a), the resulting TPA monolayer seems to be partially negatively charged. This interpretation is supported by DFT models of a deprotonated TPA adlayer, which indicate charge accumulation at the carboxylate groups compensated by screening from the metallic Ag substrate. Within the present experimental approach, the electronic characteristics of the hydrogen species cannot be unambiguously determined.

**Scheme 1:**
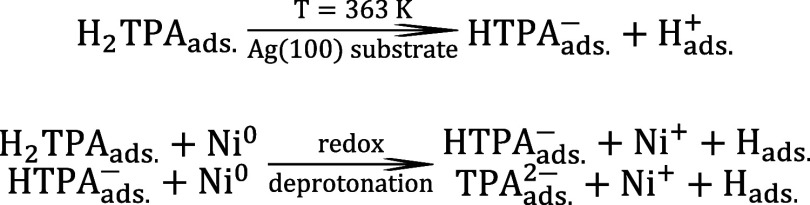
Substrate-Induced Deprotonation and Ni-Induced Redox Reactions

Subsequent Ni deposition further increases the deprotonation fraction to a maximum of 80%, corresponding to a 54% increase relative to the annealed SAM. At high Ni coverages, a weak additional contribution appears in the Ni 2p spectrum (Figure S8, light blue dots), which is assigned to Ni(0) species associated with an excessive Ni concentration and the onset of small metallic clusters. A comparison with the spectrum acquired at lower Ni coverage (Figure S9, Supporting Information) shows broadening toward lower BE, consistent with the coexistence of Ni­(I) centers and a minor Ni(0) component, rather than a shift of the Ni­(I) peak.

A systematic comparison of Ni 2p and O 1s XPS spectra for different Ni deposition doses on freshly prepared SAMs is provided in the Supporting Information (Figure S8 and Tables S3 and S4). This comparison highlights that, in analogy with previous studies,^14^ the deprotonation process becomes inhibited as the fraction approaches 70–80%. At 0.11 monolayer (ML) of Ni, a deprotonation fraction of 78% is reached despite the onset of Ni clustering. Maximum deprotonation is achieved at 0.15 ML, which is then defined as the saturation condition. Accordingly, for the samples discussed in Figures 1–3, a lower Ni coverage of 0.08 ML was used. At this dose, deprotonation is substantial while Ni(0) cluster formation remains limited, enabling reliable STM and VB measurements. Instead, for the Ni-saturated Ni-TPA chains (Figure 4) a Ni coverage of 0.15 ML is employed to induce complete deprotonation.

The Ni nodes within Ni-TPA chains are stabilized in the Ni­(I) oxidation state, as indicated by XPS results and supported by DFT simulations. This observation indicates that Ni coordination is accompanied by a redox reaction involving single-electron donation from Ni to protonated TPA molecules. Because oxidation of Ni provides only one electron per atom, this process can promote the deprotonation of a single carboxyl group out of the two available in the coordination environment (Scheme 1). Consequently, Ni coordination alone cannot fully deprotonate the TPA monolayer, resulting in a saturation deprotonation limit of approximately 50%, which should be regarded as an ensemble-averaged, experimentally constrained value rather than a strict stoichiometric rule. When combined with the approximately 30% carboxyl groups deprotonated by the Ag(100) substrate, a residual fraction of about 20% of the carboxyl groups remain protonated. Based on the experimentally determined deprotonation fraction and the stabilization of Ni­(I) inferred from XPS, two plausible coordination environments can be envisaged for Ni­(I). In one configuration, Ni­(I) is coordinated by two deprotonated −COO^–^ groups, consistent with the dominant −COO^–^ component observed in the O 1s spectra. In the other configuration, one ligand remains protonated. These configurations cannot be distinguished unambiguously by STM under the present experimental conditions, as the STM contrast is dominated by the molecular backbone and metal coordination nodes, while more subtle differences in local coordination geometry are below the accessible spatial and electronic resolution. DFT simulations suggest that the −COO^–^ group remains coordinated to Ni, whereas the interaction of the neighboring −COOH group depends on the local adsorption geometry (Supporting Information). This interaction is likely influenced by the adsorption site of Ni on Ag(100), which varies due to the incommensurate nature of the overlayer.

## Conclusions

In conclusion, by combining complementary surface-sensitive techniques, we elucidate the sequential steps governing the formation of surface-supported linear Ni-TPA chains. Following partial deprotonation induced by the substrate, subsequent Ni coordination promotes further stabilization of deprotonated carboxylate species on Ag(100) through a single-electron redox process. The stripe-like structural motif of the pristine molecular layer, defined by directional hydrogen-bonds between carboxyl groups and templated by the Ag(100) substrate, is preserved during this process, leading to well-ordered linear metal–organic chains. X-ray photoelectron spectroscopy reveals stabilization of the Ni centers in the Ni­(I) oxidation state, while valence band spectroscopy reveals coordination-induced Ni-ligand hybrid states. Complementary density functional theory calculations further support this assignment. Collectively, these results demonstrate that single-atom redox chemistry can direct the on-surface synthesis of metal–organic systems, enabling control over both structural and electronic properties in low-dimensional coordination architectures.

## Methods

### Sample Preparation

The Ag(100) crystal was cleaned using standard methods involving ionized argon (Ar^+^) sputtering and heating to 800 K. TPA was evaporated from a Knudsen cell at 410 K onto the substrate held at room temperature. Subsequently, nickel was deposited onto the SAM using a triple electron-beam evaporator (Focus GmbH) equipped with a high-purity Ni rod source (Matek GmbH, 2.0 mm diameter), while keeping the sample at 363 K. The deposition flux was monitored using a flux-monitor electrode integrated into the evaporator and maintained at a constant current of 4 nA (20 nA for the Ni reference sample). One monolayer (ML) is defined as the surface density of Ni atoms corresponding to a complete Ag(100) surface layer at saturation. Under these conditions, the Ni deposition rate was calibrated to ∼0.045 ML min^–1^, as determined from a combination of STM site-occupancy analysis and XPS measurements. All Ni coverages reported in this work are referenced to this calibration. The formation of the SAM and Ni-TPA chains was confirmed using LEED, which was also used to corroborate the Ni coverage calibration.

### STM Measurements

Scanning tunneling microscopy (STM) was performed using the commercial low-temperature STM system (CreaTec GmbH) available at the University of Graz (Austria). All images were recorded at 77 K (liquid nitrogen temperature) using electrochemically etched tungsten (*W*) tips.

### XPS Measurements

The XPS measurements were carried out at the University of Graz (Austria) using a Phoibos 100 hemispherical energy analyzer (pass energy = 10 eV, energy resolution = 0.6 eV) and a Mg K_α_ X-ray source, *h* = 1254.6 eV, with an intrinsic line width of approximately 0.7 eV (both from SPECS GmbH). The base pressure of the analysis chamber was below 2 × 10^–10^ mbar. The spectra were referenced to the Ag 3d_5/2_ peak set to a binding energy of 368.2 eV.^48^ All measurements were taken at room temperature, in a normal emission geometry, with an incidence angle of the X-ray beam of 55° from the surface normal.

Spectral fitting was performed using Voigt line shapes with the binding energies of the individual components treated as free parameters. The Lorentzian full width at half-maximum (FWHM) was fixed at 0.7 eV, consistent with core-hole lifetime broadening and the intrinsic line width of the nonmonochromatic X-ray source.^49^ A Gaussian width (*W*_G_) contribution of 0.95 eV was included to account for instrumental energy resolution and vibrational (Franck–Condon) broadening of the molecular adsorbates.^50^ The Gaussian width was optimized during the fitting procedure while being constrained to an identical value for all Voigt components. The resulting fit parameters are summarized in Tables S1–S4.

### Momentum-Resolved Photoemission Measurements

The experiments were conducted at the NanoESCA beamline of the Elettra synchrotron in Trieste, Italy, using a photoelectron emission microscope (PEEM).^51,52^ The VB photoemission spectra, band maps and work function values were obtained by setting the photon energy at 30 eV and using p-polarized light. Work-function values were extracted from the secondary-electron cutoffs. The measurements were performed at a pressure below 1 × 10^–10^ mbar with the sample cooled to 90 K using an open-cycle cryostat. The total energy resolution was 100 meV and the momentum resolution of the PEEM was ±0.05 Å^–1^. To avoid radiation damage on the molecular-based frameworks, the samples were rastered during measurements.

## Supplementary Material



## Notes

Additional structural characterization of the TPA self-assembled layer and Ni-TPA chains by LEED and STM, as well as supplementary valence band and XPS spectra. A momentum map of the clean Ag(100) surface, indicating the high-symmetry points of the surface Brillouin zone, is also provided. Tables summarizing the results of the Voigt fits applied to all XPS spectra are included. Theoretical modeling of the TPA self-assembled layer and Ni-TPA chains is also described (PDF)

## ∇

Physical and Computational Sciences Directorate and Institute for Integrated Catalysis, Pacific Northwest National Laboratory, Richland, Washington 99354, United States

## Abbreviations

TPA: terephthalic acid
TM: transition metal
TCNB: 1,2,4,5-tetracyanobenzene
TCNQ: 7,7,8,8-tetracyanoquinodimethane
SAM: self-assembled monolayer
MOF: metal–organic frameworks
MOC: metal–organic chains
HOMO: highest occupied molecular orbital
ML: monolayer
DFT: density functional theory
