# Supplementary material for: Single Ni Atoms Drive Carboxyl Deprotonation in Metal–Organic Chains
Source: ACS Nano. 2026 Apr 17;20(16):12596–603. doi: 10.1021/acsnano.6c01444 (PMC13131048; doi:10.1021/acsnano.6c01444)
Supplement: Supplementary file 1 [file nn6c01444_si_001.pdf]

Supporting information

Single Ni Atoms Drive Carboxyl Deprotonation in Metal-Organic Chains

Simone Mearini,[a] Fabian Auer,[b] Maximilian Laßhofer,[b] Andreas Windischbacher,[b]*

Dominik Brandstetter,[b] Daniel Baranowski,[a,†] Yan Yan Grisan Qiu,[a] Iulia Cojocariu,[c,d]

Matteo Jugovac,[c,d] Martin Sterrer,[b] Giovanni Zamborlini,[b]

Vitaliy Feyer, [a,e] and Claus Michael Schneider[a,e,f]*

[a] Peter Grünberg Institute (PGI-6), Jülich Research Centre, 52428 Jülich, Germany

[b] Institute of Physics, NAWI Graz, University of Graz, 8010 Graz, Austria

[c] Physics Department, University of Trieste, 34127 Trieste, Italy

[d] Elettra - Sincrotrone Trieste S.C.p.A., S.S. 14 km 163.5, 34149 Trieste, Italy

[e] Faculty of Physics and Center for Nanointegration Duisburg-Essen (CENIDE), University of Duisburg-Essen, 47048 Duisburg, Germany

[f] Department of Physics and Astronomy, UC Davis, Davis CA 95616, USA

Corresponding Author

*s.mearini@fz-juelich.de, v.feyer@fz-juelich,de

Present Addresses

†Physical and Computational Sciences Directorate and Institute for Integrated Catalysis, Pacific Northwest National Laboratory, Richland, Washington 99354, USA

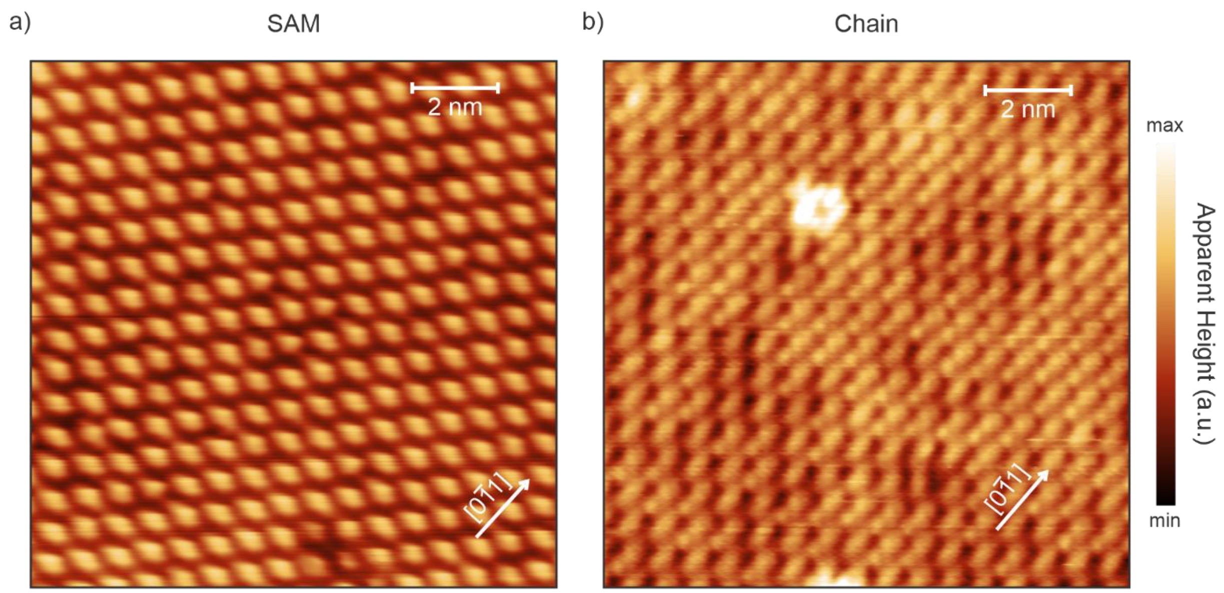

Figure S1. Large-scale STM images of a) the SAM "α-phase" (12.2 × 12.2 nm², -3.3 V, 0.5 nA) and b) the Ni-TPA chains (12.2 × 12.2 nm², -0.1 V, 0.5 nA).

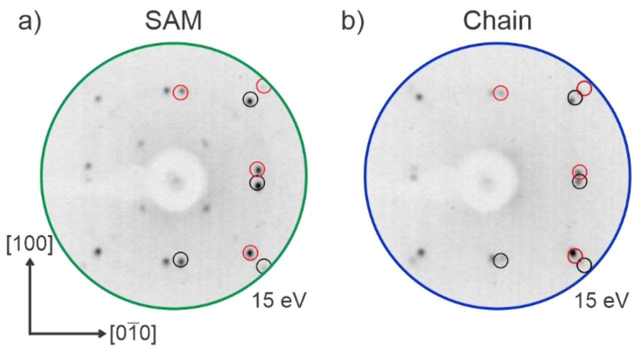

Figure S2. LEED pattern acquired with an incident electron beam energy of 15 eV kinetic energy for a) the SAM and b) the Ni-TPA chains. The superimposed black and red circles represent the two rotational domains of the simulated pattern, obtained for a unit cell with matrices a) (2, 1.7; 2, -1.7) and b) (1.9, 1.7; 1.9, -1.7), respectively.

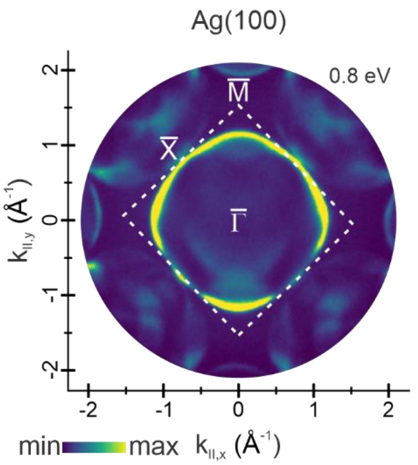

Figure S3. Experimental 2D momentum map (hν = 30 eV, p-polarized light) acquired for the clean Ag(100) substrate at 0.8 eV BE. $\bar{M}$, $\bar{\Gamma}$ and $\bar{X}$ indicate the locations of the high-symmetry points in the surface Brillouin zone.

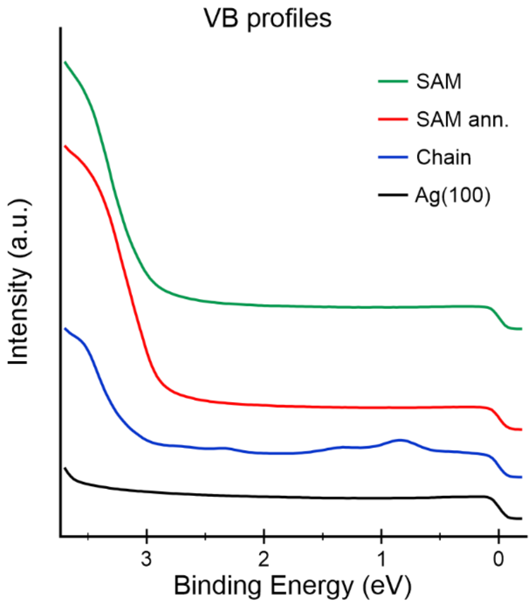

Figure S4. Experimental momentum-integrated VB spectra (hν = 30 eV, p-polarized light) acquired for the clean Ag(100) surface, the SAM, the annealed layer (T = 363K), and the Ni-TPA chains within the BE range of [-0.2; 3.7] eV. The four systems are shown as black, green, red and blue curves, respectively.

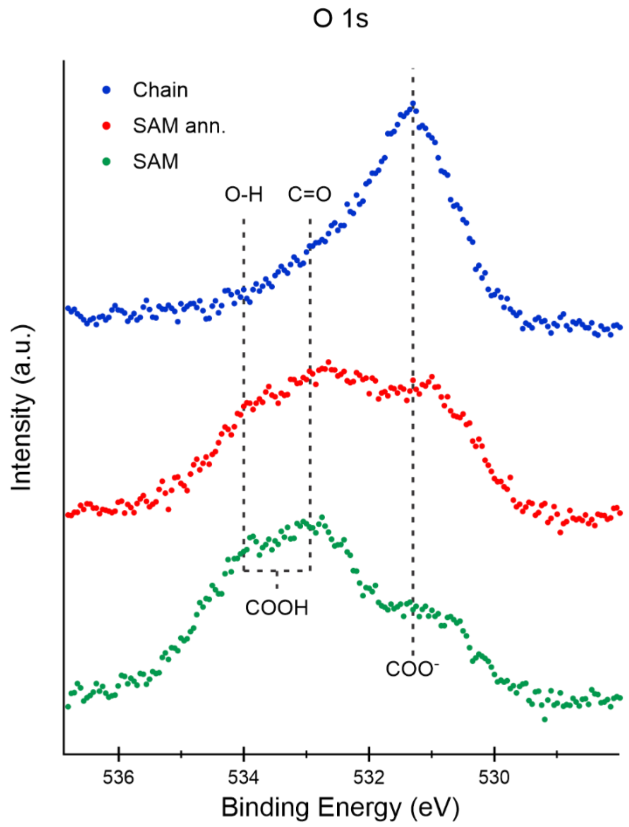

Figure S5. O 1s XPS spectra (dots) for the SAM, the annealed layer (T = 363 K) and the Ni-TPA chains, represented by the green, red and blue dots, respectively.

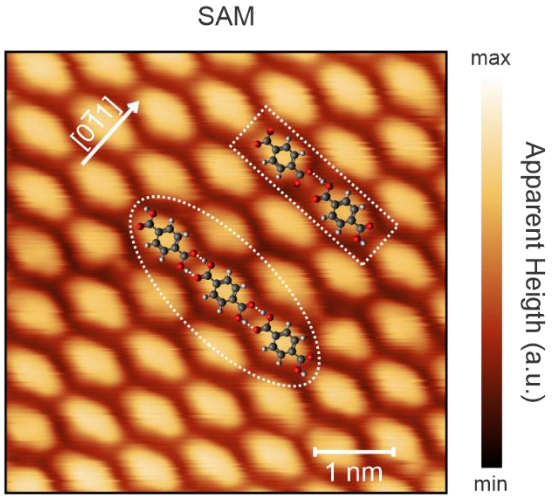

Figure S6. STM image of the SAM (5.5 × 5.5 nm², -3 V, 0.5 nA). The dashed white ellipse encloses the model of the SAM molecular arrangement, while the dashed white rectangle highlights a defect due to the presence of a TPA^{-}/TPA^{2-} molecule forming a single hydrogen-bond.

Table S1. Results of the Voigt line-shape fits applied to the O 1s XPS spectra of the SAM and the Ni-TPA chains. The Lorentzian and Gaussian contributions were described by full widths at half-maximum (FWHM) of 0.70 eV and 0.95 eV, respectively.

		COOH		
Networks	*O 1s*	*O-H*	*C=O*	*COO^{-}*
SAM	*BE (eV)*	*533.8*	*532.6*	*531.0*
	Area	*39%*	*39%*	*22%*
Chain, 0.08 ML Ni	*BE (eV)*	*533.1**	*532.2**	*531.2***
	Area	*14%*	*14%*	*72%*

* single hydrogen-bond

** Ni interaction

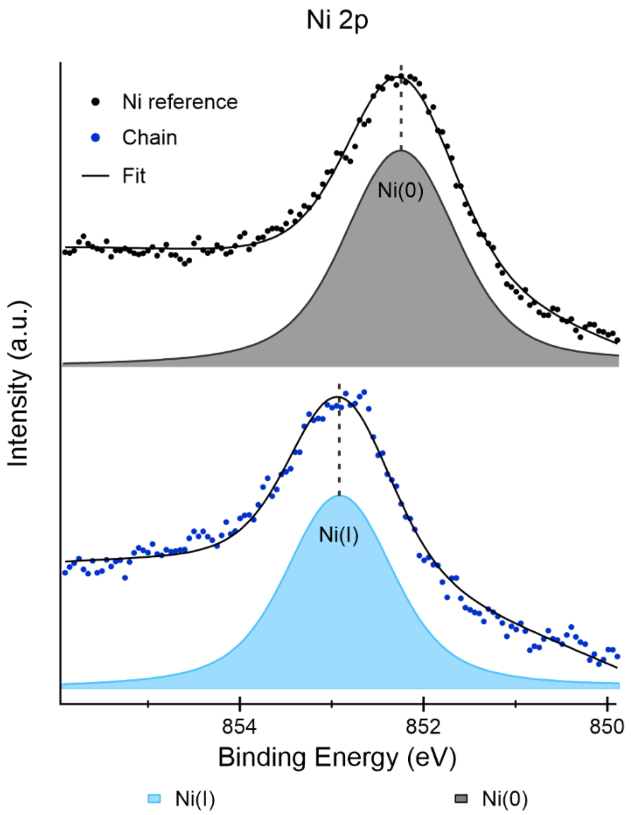

Figure S7: Ni 2p XPS spectra (dots) and corresponding fits (black curves) using Voigt functions of the Ni-TPA chains (0.08 ML, blue) and a Ni reference (0.38 ML, black); the deconvolution is reported in cyan and gray for the Ni(I) and Ni(0) atoms.

Table S2. Results of the Voigt line-shape fits applied to the O 1s XPS spectra for (a) the freshly prepared SAM, (b) the annealed layer (T = 363 K), and (c) the Ni-TPA chains. Lorentzian and Gaussian FWHM values were fixed to 0.70 eV and 0.95 eV, respectively.

		COOH		
Networks	*O 1s*	*O-H*	*C=O*	*COO^-*
SAM	*BE (eV)*	*533.8*	*532.6*	*531.0*
	Area	*42%*	*42%*	*16%*
SAM ann., T = 363 K	*BE (eV)*	*533.7**	*532.4**	*531.0*
	Area	*37%*	*37%*	*26%*
Chain, 0.15 ML Ni	*BE (eV)*	*533.1**	*532.2**	*531.2*
	Area	*10%*	*10%*	*80%*

*mixed contributions from single- and double-hydrogen-bonded -COOH groups

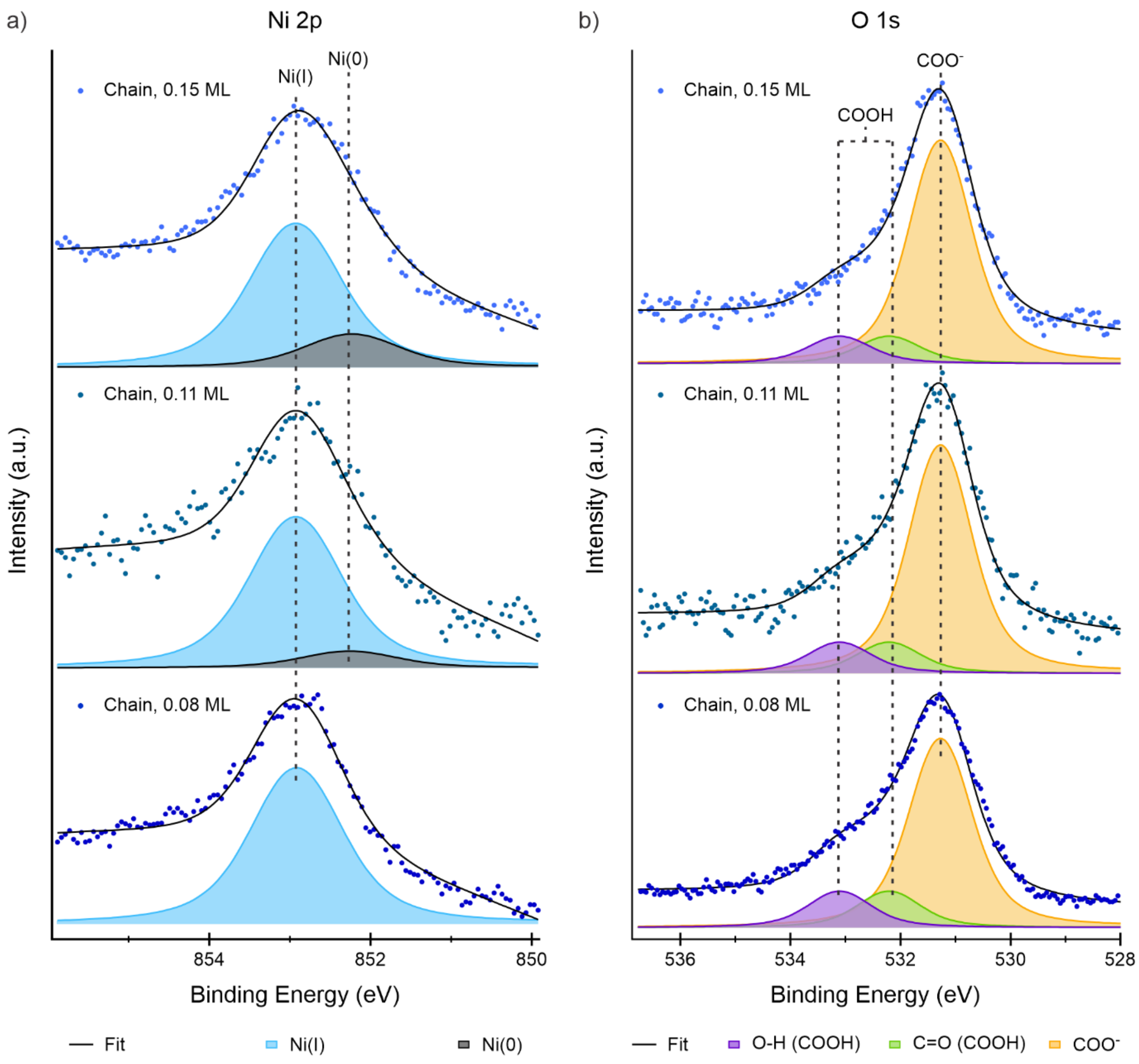

Figure S8. a) Ni 2p and b) O 1s XPS spectra (dots) and corresponding fits (black curves) using Voigt functions for Ni-TPA chains formed with different Ni concentrations (0.08 to 0.15 ML); the chains spectra are shown in blue, teal and light blue for increasing Ni amounts. In a) the deconvolution is reported in cyan and gray for the Ni(I) and Ni(0) atoms, respectively, and the intensity is rescaled by a factor 0.75 and 0.5 for the systems with Ni concentrations of 0.11 ML and 0.15 ML, respectively. In b) the deconvolution is reported in purple and light green for the O-H and C=O oxygen atoms from the -COOH groups, respectively, and in orange for the COO^- oxygen atoms in TPA^{2-}.

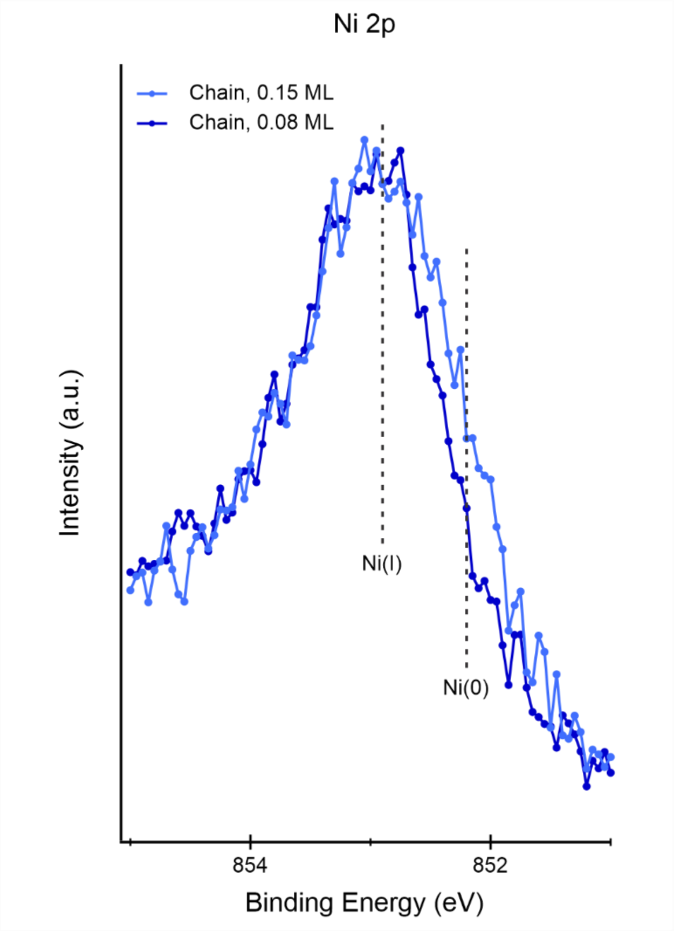

Figure S9. Ni 2p spectra for Ni-TPA chains formed with Ni concentrations equal to 0.08 (blue) and 0.15 ML (light blue, intensity rescaled by a factor 0.5).

Table S3. Results of the Voigt fits applied to the Ni 2p XPS spectra shown in Figure S8 and to a Ni reference layer (0.38 ML). Lorentzian and Gaussian FWHM values were fixed to 0.70 eV and 0.95 eV, respectively.

Networks	*Ni 2p*	*Ni(I)*	*Ni(0)*
Chain,	*BE (eV)*	*852.9*	*-*
0.08 ML Ni	*Area*	*100%*	
Chain,	*BE (eV)*	*852.9*	*852.2*
0.11 ML Ni	*Area*	*90%*	*10%*
Chain,	*BE (eV)*	*852.9*	*852.2*
0.15 ML Ni	*Area*	*81%*	*19%*
Ni ref.,	*BE (eV)*	*-*	*852.2*
0.38ML	*Area*		*100%*

Table S4. Results of the Voigt line-shape fits applied to the O 1s XPS spectra of the samples reported in Figure S8. Lorentzian and Gaussian FWHM values were fixed to 0.70 eV and 0.95 eV, respectively.

		COOH		
Networks	*O 1s*	*O-H*	*C=O*	*COO⁻*
Chain,	*BE (eV)*	*533.1*	*532.2*	*531.2*
0.08 ML Ni	*Area*	*14%*	*14%*	*72%*
Chain,	*BE (eV)*	*533.1*	*532.2*	*531.2*
0.11 ML Ni	*Area*	*11%*	*11%*	*78%*
Chain,	*BE (eV)*	*533.1*	*532.2*	*531.2*
0.15 ML Ni	*Area*	*10%*	*10%*	*80%*

Theoretical Models

To corroborate our experimental data and gain further insight into the deprotonation of TPA before and after Ni deposition, we performed density functional theory (DFT) calculations for TPA/Ag and Ni-TPA/Ag interfaces.

Modelling a full interface within a periodic slab approach is, however, challenging in this system. The incommensurate nature of both the TPA-SAM and the Ni-TPA overlayer on Ag(100) would require prohibitively large supercell to achieve a realistic description even at the level of GGA-type DFT. In addition, a comprehensive exploration of potential energy landscapes of hydrogen species related to the deprotonation process on the surface is beyond the scope of this work.

To address these limitations while retaining reasonable conclusions, we adopted a simplified interface model based on a tweaked, clean [3, -2; 3, 2] epitaxy (Figure S10a, inset), which allows us to simultaneously model periodic TPA chains and probe different H adsorption sites. A smaller unit cell, e.g. [2, -2; 2, 2], would better capture inter-chain interactions but would not provide enough to accommodate H in different adsorption sites.

For the pristine TPA monolayer on Ag(100), we considered two scenarios: (i) fully protonated H_2TPA molecule adsorbed on the surface, and (ii) singly deprotonated HTPA molecule with a lone H atom adsorbed nearby, representing the coexistence observed in the SAM based on the XPS analysis. In both cases, the molecule adsorbs preferentially flat, with the benzene ring lying above a hollow site of the Ag(100) surface (Figure S10a, b). For H_2TPA, the projected density of states (Figure S10c) shows no evidence of charge transfer between the molecule and the substrate, with the HOMO located at approximately 3 eV binding energy, in good agreement with the experimental data (Figure 2a).

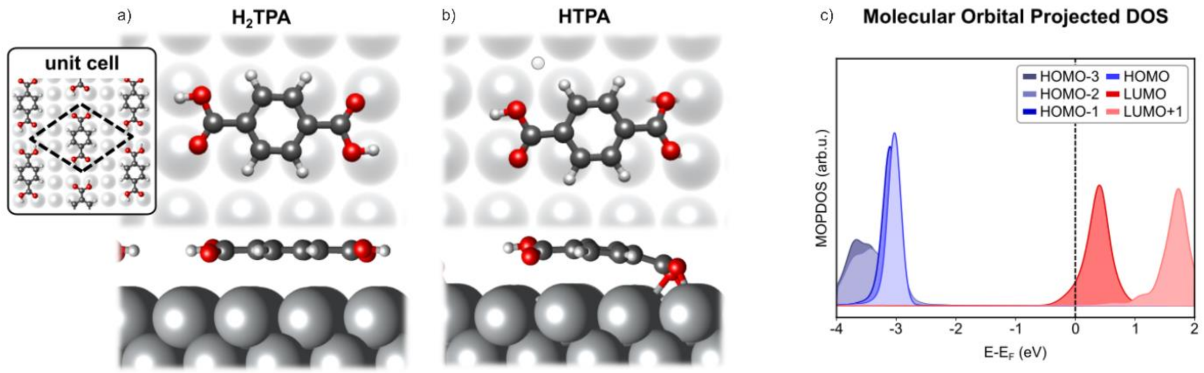

Figure S10. a, b) Top and side views of the surface-supported real-space structural models of a) a fully protonated H_2TPA molecule and b) a singly deprotonated HTPA molecule with a nearby H atom; c) DOS projected onto selected molecular orbitals for the structural model in a).

Upon deprotonation, the carboxylate group bends towards the surface, while the H preferentially occupies a hollow-site (see Figure S10b), consistent with previous studies of H adatoms on Ag(100).[1,2] Analysis of the charge density distribution suggests that the carboxylate group of the HTPA remains negatively charged, with this charge compensated by screening from the Ag substrate. DFT calculations further show that deprotonation on the surface is energetically unfavorable by 0.44 eV compared to the H_2TPA/Ag system, consistent with the experimentally observed partial deprotonation (approximately 10-20% prior to annealing).

The calculations also reproduce the negligible change in the experimental work-function (WF) reported in Table S5. In the presence of only physisorbed H_2TPA species, the WF decreases by -0.31 eV relative to clean Ag(100) due to the Pauli-pushback effect.[3,4] In contrast, a partially deprotonated monolayer (50% in the model) leads to an increase by +0.37 eV in WF, reflecting the contribution of the bent $-COO^-$ groups and associated charge redistribution and molecular dipoles. At the experimentally observed deprotonation level (about 30%), these opposing effects largely compensate, resulting in minimal net WF change.

Table S5. Work function (Φ) values determined as the difference between the framework and the Ag(100) values, respectively, for the systems studied in Figures 1-3.

Networks	*ΔΦ (eV)*
Ag(100)	*0.0*
SAM	*0.0*
Chain, 0.08 ML Ni	*+ 0.1*

For Ni incorporation into the TPA chains, we considered two coordination environments: Ni coordinated between a protonated and a deprotonated TPA (HTPA-Ni, Figure S11a), and Ni coordinated to two deprotonated TPA units (TPA-Ni, Figure S11b). In both cases, the projections onto the Ni d-states (Figure S11a, b) indicate stabilization of Ni in the Ni(I) oxidation state, i.e. a d^9 configuration, consistent with the redox process inferred from XPS. Furthermore, both systems exhibit molecular states extending up to 1 eV binding energy, consistent with the experimental momentum-integrated valence band data (Figure 2c) and indicative of Ni-TPA hybridization.

A closer inspection of the HTPA-Ni structure shows that Ni relaxes toward hollow sites of the Ag(100) surface, independently of the starting position, and resides slightly below the molecular plane. The Ni atom coordinates primarily to the deprotonated carboxylate group, while forming weaker interactions with the protonated group. To verify that this behavior is not an artifact of the chosen unit cell, we performed additional calculations for freestanding layers, i.e. without the substrate, using both the [3, -2; 3, 2] unit cell and the experimentally-derived incommensurate lattice parameters. In both cases, the molecules rotate during relaxation in the absence of the substrate, while Ni remains within the molecular plane and positions itself approximately equidistant between the two TPA units (Figure S11c). This indicates that the asymmetry observed

in the supported model originates from molecular-substrate interactions. In all cases, including the freestanding configurations, Ni remains stabilized in a Ni(I) d^9 configuration.

In the fully-deprotonated supported model (TPA-Ni), Ni is lifted away from the surface, leading to the formation of chain-like MOF structures with slight in-plane molecular rotation off the chain direction. The associated charge redistribution introduces an additional dipole, resulting in a calculated WF increase of +0.2 eV, in good agreement with the experimentally observed value of +0.1 eV.

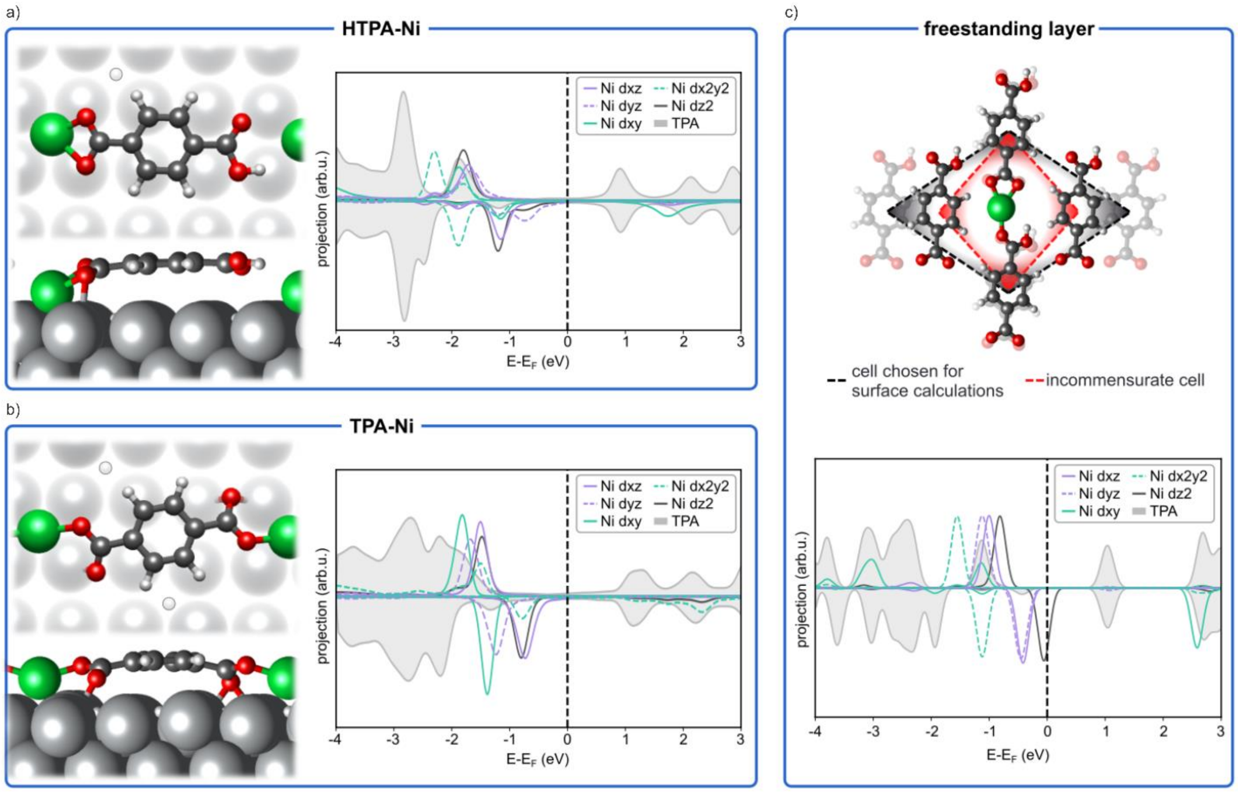

Figure S11. a, b) Top and side views of the real-space structural models (left) and DOS (right) projected onto individual Ni d states (curves) and TPA molecular orbitals (shaded areas) for a) HTPA-Ni and b) TPA-Ni models. c) Real-space structural model (top) and DOS (bottom) projected onto singular Ni d states (curves) and TPA molecular orbitals (shaded area) for the freestanding model.

Theoretical Details

Density functional theory calculations were performed using VASP 6.4 within a repeated slab approach, employing at least 20 Å of vacuum layer. The geometry was fully relaxed on a 6x6x1 k-mesh until forces dropped below 0.01 Å-1 using the PBE functional and a self-interaction error correction in the Dudarev ansatz (Ueff = 3 eV). The final density of states calculation employed a tighter k-mesh on a 10 x 10 x 1 Gamma centred grid.

References

(1) Gómez, E. del V.; Amaya-Roncancio, S.; Avalle, L. B.; Linares, D. H.; Gimenez, M. C. DFT Study of Adsorption and Diffusion of Atomic Hydrogen on Metal Surfaces. *Appl. Surf. Sci.* **2017**, *420*, 1–8. DOI: 10.1016/J.APSUSC.2017.05.032.

(2) Gómez, E. del V.; Sanchez-Varretti, F. O.; Avalle, L. B.; Ramirez-Pastor, A. J.; Gimenez, M. C. Entropy Study of Adsorption of H and O Atoms on Ag(001) and Cu(001) Surfaces, by Means of DFT, Monte Carlo Simulations and Cluster Approximation. *Phys. A* **2025**, *674*, 130693. DOI: 10.1016/J.PHYSA.2025.130693.

(3) Cornil, D.; Van Regemorter, T.; Beljonne, D.; Cornil, J. Work Function Shifts of a Zinc Oxide Surface upon Deposition of Self-Assembled Monolayers: A Theoretical Insight. *Phys. Chem. Chem. Phys.* **2014**, *16* (38), 20887–20899. DOI: 10.1039/C4CP02811B.

(4) Hofmann, O. T.; Rangger, G. M.; Zojer, E. Reducing the Metal Work Function beyond Pauli Pushback: A Computational Investigation of Tetrathiafulvalene and Viologen on Coinage Metal Surfaces. *J. Phys. Chem. C* **2008**, *112* (51), 20357–20365. DOI: 10.1021/jp806834g.
